# Polymorphisms of Serotonin Receptor 2A and 2C Genes and *COMT* in Relation to Obesity and Type 2 Diabetes

**DOI:** 10.1371/journal.pone.0006696

**Published:** 2009-08-19

**Authors:** Sofia I. I. Kring, Thomas Werge, Claus Holst, Søren Toubro, Arne Astrup, Torben Hansen, Oluf Pedersen, Thorkild I. A. Sørensen

**Affiliations:** 1 Institute of Preventive Medicine, Copenhagen University Hospital, Centre for Health and Society, Copenhagen, Denmark; 2 Center for Pharmacogenomics, the Panum Institute, University of Copenhagen, Copenhagen, Denmark; 3 Research Institute of Biological Psychiatry, Sct. Hans Hospital, University of Copenhagen, Copenhagen, Denmark; 4 Reduce – a Research Clinic, Roskilde, Denmark; 5 Department of Human Nutrition, Centre of Advanced Food Research, Faculty of Life Sciences, University of Copenhagen, Frederiksberg, Denmark; 6 Hagedorn Research Institute and Steno Diabetes Center, Gentofte, Denmark; 7 Faculty of Health Science, University of Southern Denmark, Odense, Denmark; 8 Institute of Biomedicine, Faculty of Health Science, University of Copenhagen, Copenhagen, Denmark; 9 Faculty of Health Science, University of Aarhus, Aarhus, Denmark; University of Camerino, Italy

## Abstract

**Background:**

Candidate genes of psychological importance include *5HT2A, 5HT2C*, and *COMT*, implicated in the serotonin, noradrenaline and dopamine pathways, which also may be involved in regulation of energy balance. We investigated the associations of single nucleotide polymorphisms (SNPs) of these genes with obesity and metabolic traits.

**Methodology/Principal Findings:**

In a population of 166 200 young men examined at the draft boards, obese men (n = 726, BMI≥31.0 kg/m^2^) and a randomly selected group (n = 831) were re-examined at two surveys at mean ages 46 and 49 years (S-46, S-49). Anthropometric, physiological and biochemical measures were available. Logistic regression analyses were used to assess age-adjusted odds ratios. No significant associations were observed of *5HT2A* rs6311, *5HT2C* rs3813929 and *COMT* rs4680 with obesity, except that *COMT* rs4680 GG-genotype was associated with fat-BMI (OR = 1.08, CI = 1.01–1.16). The SNPs were associated with a number of physiological variables; most importantly *5HT2C* rs3813929 T-allele was associated with glucose (OR = 4.56, CI = 1.13–18.4) and acute insulin response (OR = 0.65, CI = 0.44–0.94) in S-49. *COMT* rs4680 GG-genotype was associated with glucose (OR = 1.04, CI = 1.00–1.09). Except for an association between *5HT2A* rs6311 and total-cholesterol at both surveys, significant in S-46 (OR = 2.66, CI = 1.11–6.40), no significant associations were observed for the other phenotypes. Significant associations were obtained when combined genotype of *5HT2C* rs3813929 and *COMT* rs4680 were examined in relation to BMI (OR = 1.12, CI = 1.03–1.21), fat-BMI (OR = 1.22, CI = 1.08–1.38), waist (OR = 1.13, CI = 1.04–1.22), and cholesterol (OR = 5.60, CI = 0.99–31.4). Analyses of impaired glucose tolerance (IGT) and type 2 diabetes (T2D) revealed, a 12.3% increased frequency of *5HT2C* rs3813929 T-allele and an 11.6% increased frequency of *COMT* rs4680 GG-genotype in individuals with IGT or T2D (χ^2^, p = 0.05 and p = 0.06, respectively). Examination of the combined genotypes of *5HT2C* and *COMT* showed a 34.0% increased frequency of IGT or T2D (χ^2^, p = 0.01).

**Conclusions:**

The findings lend further support to the involvement of serotonin, noradrenaline and dopamine pathways on obesity and glucose homeostasis, in particular when combined genotype associations are explored.

## Introduction

The fundamental causes of obesity relate to a complex interplay between environmental exposure, health behaviour and inherited components [1]. Obesity and body fat distribution are important determinant of glucose homeostasis [2], [3] with insulin resistance as the main contributor to the deleterious consequences of obesity, such as development of metabolic diseases, IGT, T2D and eventually cardiovascular disease [4]. Deeper insights into the genetic control of energy metabolism are warranted in order to improve long-term compliance to prevention programs or therapeutic interventions.

Recently, genes with known psychophysiological functions have gained much attention in the field of obesity and a common pathophysiology have been suggested between depression, obesity and related metabolic disorders [5]–[7]. The neurotransmitters, serotonin, noradrenaline and dopamine are important in the central nervous system regulation of many physiological processes including energy and glucose homeostasis [8]–[10]. Consequently, these neural pathways are interesting targets for anti-obesity drugs, antidepressants and agents for treatment of various eating disorders [10]–[12]. However, major drug-induced adverse events include body weight gain in treatment of psychiatric disorders [10], [13] and mood disorders in anti-obesity agents [14]. The large intra- and inter-individual variability in clinical response to antipsychotic drugs and their side effects is further linked to genetic susceptibility [10], [11], [13]. Pharmacogenetic research focuses therefore on identification of specific genes that influence drug response by utilizing a candidate-gene approach [10], [11], although supporting evidence for a role of these genes in development of obesity and associated metabolic traits from population-based observational studies is sparse.

A plethora of studies indicates that serotonin (5-hydroxytryptamine; 5HT) receptor 2A *(5HT2A)* and 2C *(5HT2C)* are involved in the regulation of appetite and energy homeostasis [15], although with functional differences between the genes [16]. Thus, *5HT2C* -759C/T is shown to affect treatment-induced weight gain in schizophrenic patients [17], and *5HT2A* –1438G/A has been linked to several neuro-psychiatric disorders [18]–[20] and to abdominal obesity [21], [22]. Transcripts of the serotonin receptor have been found in the hypothalamus, including in the para-ventricular nucleus, lesions of which result in obesity [23].

Catechol-O-methyltransferase *(COMT)* encodes for the COMT enzyme responsible for the degradation of the catecholamines, dopamine adrenaline and noradrenaline through both central and peripheral effects [24], [25]. There is evidence that changes in the activity of COMT affect sympathetic tone, regulate the amounts of active dopamine and noradrenaline in various cerebral regions and thus is involved in multiple reward-motivated behaviour such as obesity [10], [11], [24], [25], mood and other mental processes [11], [26]. Several polymorphisms of *COMT* have been described, but 24938A/G (rs4680) is the most extensively studied [26] showing associations with schizophrenia, alcoholism and obesity [27].

In the present study, we examined *5HT2A* –1438G/A (rs6311), *5HT2C* -759C/T (rs3813929) and *COMT* 24938 A/G (rs4680) in relation to obesity and related metabolic quantitative traits in a group of middle-aged men representing a very broad range of BMI.

## Methods

### Study population

Among 362 200 Caucasian men examined at the mean age of 20 years at the draft boards in Copenhagen and its surroundings during 1943–77, a randomly selected group of one in every hundred men (n = 3 601) and all obese men (n = 1 930) were manually identified. Obesity was defined as 35% overweight relative to a local standard in use at the time, and this corresponds to a BMI≥31.0 kg/m^2^, which proved to be above the 99th percentile. All obese and half of the random sample, still living in the region, were invited to a follow-up survey in 1992–94 at the mean age of 46 years (survey S-46) and in 1998–2000 at the mean age of 49 years (survey S-49). The criteria for invitation to the follow-up surveys and the participation have been described previously [28]–[30], and the number of participants shows the expected attrition over time (Table 1). Phenotypic assessments were carried out at both surveys, though much more thoroughly at the second survey (S-49), and DNA was extracted from blood sample buffy coats at the S-46. In total, 1 557 (726 obese and 831 randomly selected) participants were genotyped for the present study, indicating that the randomly selected group represent 166 200 men originally identified at the draft board examination. Among these 485 (209 obese and 276 randomly selected) had been assessed in S-49.

**Table 1:** Genotype distribution (%) and minor allele frequencies for the examined genes and SNPs in obese and controls participants.

Survey	Gene	Chromosomal location	dbSNP	SNP^1^	Obese				Controls			
					Genotype			MAF	Genotype			MAF
					*Wt*	*He*	*Ho*		*Wt*	*He*	*Ho*	
S-46	*5HT2A*	13q14-q21	Rs6311	G/**A**	249 (34.6)	375 (52.4)	93 (13.0)	0.39	320 (38.8)	378 (46.0)	125 (15.2)	0.38
	*5HT2C*	Xq24	Rs3813929	C/**T**	613 (84.5)	−	113 (15.5)	0.16	695 (83.7)	−	136 (16.3)	0.16
	*COMT*	22q11.21–q11.23	Rs4680	A/**G**	237 (33.0)	335 (46.7)	146 (20.3)	0.43	265 (32.4)	397 (48.6)	154 (19.0)	0.43
S-49	*5HT2A*	13q14–q21	Rs6311	G/**A**	70 (34.2)	111 (54.1)	24 (11.7)	0.39	107 (39.2)	125 (45.8)	41 (15.0)	0.40
	*5HT2C*	Xq24	Rs3813929	C/**T**	175 (83.7)	−	34 (16.3)	0.16	237 (85.9)	−	39 (14.1)	0.14
	*COMT*	22q11.21–q11.23	Rs4680	A/**G**	64 (31.4)	103 (50.5)	37 (18.1)	0.43	80 (29.5)	142 (52.4)	49 (18.1)	0.44

1 The minor alleles are shown in bold-faced letters. SNPs = single nucleotide polymorphisms, Wt = wild type, He = heterozygote, Ho = homozygote, MAF = minor allele frequency.

We certify that all applicable institutional and governmental regulations concerning the ethical use of human volunteers were followed during this research. The Danish Data Protection Agency and the regional Ethical Committee approved the study to be in accordance with the Helsinki Declaration II. All participants signed a written consent before participating.

### Phenotypic measurement

Waist circumference (cm) was measured according to the WHO recommendations [31] to the nearest 0.5 cm with the subjects standing, using a nonexpendable linen tape measure,. Total body fat mass (kg) was assessed by bioimpedance at S-46 and from the DEXA scan at S-49. Fat body mass index (fat-BMI; kg/m^2^) was calculated as total body fat mass (kg) divided by height (m) squared.

Participants in S-46 had non-fasting glucose and lipid levels determined on fresh plasma samples. In the S-49 cohort, an oral glucose tolerance test (OGTT) was conducted, except in individuals with diagnosed and therefore likely treated diabetes (n = 10) [28]. Classification of IGT or T2D was done according to the WHO diagnostic criteria [32]. Insulin sensitivity and acute insulin response were assessed by the recently recommended BIGTT indices (BIGTT-S_I_ and BIGTT-AIR, respectively) on the basis of measurements of plasma glucose and serum insulin at the time points 0, 30 and 120 minutes during the OGTT [33]. Details on data collections and measurement of anthropometric and other phenotypic estimates have been described elsewhere [28], [34], [35].

### Molecular genetic analyses

Genotyping of *5HT2A* -1438G/A (rs6311), *5HT2C* -759C/T (rs3813929), and *COMT* 24938A/G (rs4680) was performed using Taqman allelic discrimination (KBioscience, Herts, UK). Genotype data were obtained in 96.7–98.7% of the DNA samples with a genotype error rate of 0.0%.

### Statistics

A likelihood ratio test for an additive, a dominant and a recessive effect of the genotyped SNPs determined that a dominant genetic model was chosen for *5HT2A* rs6311 and (wild type versus heterozygous and homozygous genotype), and a recessive genetic model was chosen for the present analyses of *COMT* rs4680 (wild type and heterozygous genotype versus homozygous genotype). The *5HT2C* receptor gene is located at the X chromosome, and therefore the men in the present study had either one C- or T-allele.

In order to properly take into account and optimally utilise the strength of the sampling design, the two groups of obese and controls have been analysed together at each follow-up survey S-46 and S-49. The massive enrichment of the right tail of the BMI distribution implies that the data cannot be analysed with BMI or BMI-associated outcomes as response variables in common regression models. Using a dichotomized case-control approach would waste considerable statistical efficiency otherwise gained by using the quantitative phenotypes. Limiting the analyses to obese and control groups would also be a waste of the efficiency that is implicit in the sampling design and would disregard the point in having a sampling from a study population of 166,200 subjects at S-46 and 55,200 subjects at S-49. The greater statistical power of the wide coverage of the quantitative phenotypes was gained by reversing the statistical models for the associations, i.e. estimation of the odds of carrying the particular risk-allele genotype for a given level of the phenotypes. This can be done without distributional assumptions about the phenotypes. Standard logistic regression analysis was applied to assess the odds ratios (ORs) of the genotype (response variable) in relation to the phenotypes (covariates, harmonised as z-scores for obesity phenotypes) in the combined case and control groups. The regression model includes relevant phenotypic predictors of this response. Logistic regressions have previously been applied to genetic association studies in which alleles or genotypes are treated as dependent variables [36]–[41]. All analyses were adjusted for age as a continuous variable. P-values<0.05 were considered statistically significant. Implications of multiple testing are addressed in the discussion. Analyses were performed using SAS statistical procedures (version 9.1; SAS Institute Inc, Cary, NC).

## Results

The distribution of the genotypes was almost the same at S-46 and S-49 indicating that the attrition of the study groups by time was not dependent on the genotype **(** **Table 1** **)**. Hardy-Weinberg equilibrium for each SNP was tested in controls and revealed no significant departures.

### BMI, fat-BMI and waist circumference

We estimated the ORs of the genotype (response variable) in relation to the phenotypes (covariates). Results from logistic regression analyses for obesity phenotypes are given in odds ratios with 95% confidence intervals (CIs).

Results from the logistic regression analyses showed that neither *5HT2A* –1438G/A nor *5HT2C –759C/T* was significantly associated with BMI, fat-BMI or waist circumference **(** **Table 2** **)**. The *COMT* 24938A/G was associated with fat-BMI at S-46, where a two-unit increase in fat-BMI increased the odds for the GG-genotype by 8% (OR = 1.08, CI = 1.01–1.16, p = 0.03). *COMT* 24938A/G was associated with waist circumference at S-46 with borderline significance, where an increment of five cm in waist circumference increased the odds for the GG-genotype by 4% (OR = 1.04, CI = 1.00–1.09, p = 0.06).

**Table 2:** Odds ratio (OR) including 95% confidence intervals (CI) for *5HT2A* rs6311 (dominant effect of the A allele, AA & AG *vs* GG), *5HT2C* rs3813929 (effect of the T *vs* C alleles) and *COMT* rs4680 (recessive effect of the G allele, GG *vs* GA & AA) in relation to obesity phenotypes.

	*5HT2A*		*5HT2C*		COMT	
	-1438G/A		-759C/T		24938A/G	
Obesity phenotypes	OR (95% CI)	*P*	OR (95% CI)	*P*	OR (95% CI)	*P*
*S-46 (n = 1570)*						
BMI (kg/m^2^)^1^	1.01 [0.97; 1.04]	0.70	1.02 [0.97; 1.07]	0.40	1.03 [0.99;1.08]	0.13
Fat-BMI (kg/m^2^)^1^	0.99 [0.93; 1.05]	0.73	1.04 [0.96; 1.12]	0.31	1.08 [1.01; 1.16]	0.03
Waist (cm)^2^	1.00 [0.97; 1.04]	0.81	1.03 [0.98; 1.07]	0.29	1.04 [1.00; 1.09]	0.06
S-49* (n = 551)						
BMI (kg/m^2^)^1^	1.00 [0.95; 1.06]	0.99	1.03 [0.96; 1.11]	0.41	1.03 [0.96; 1.10]	0.47
Fat-BMI (kg/m^2^)^1^	1.01 [0.93; 1.10]	0.82	1.07 [0.95; 1.20]	0.25	1.04 [0.94; 1.16]	0.43
Waist (cm)^2^	1.01 [0.95; 1.03]	0.80	1.04 [0.97; 1.12]	0.26	1.02 [0.95; 1.09]	0.55

BMI = body mass index, fat-BMI = fat body mass index.

1 Per two-unit increment of BMI or fat-BMI.

2 Per five cm increment of waist circumference.

### Metabolic traits

There were several statistically significant associations between the examined SNPs and some of the metabolic traits. *5HT2A* –1438G/A was associated with serum cholesterol at S-46, where a one-unit (10 mmol/L) increase in cholesterol increased the odds for the minor A-allele 2.7 fold (OR = 2.66, CI = 1.11–6.40, p = 0.03), and an almost similar estimate was seen at S-49, albeit not significant **(** **Table 3** **)**. *5HT2C* –759C/T was associated with non-fasting plasma glucose at S-49, for which a one-unit (10 mmol/L) increase in glucose increased the odds for the TT-genotype 4.6 fold (OR = 4.56, CI = 1.13–18.4, p = 0.03). Likewise, a one-unit increase in acute insulin response decreased the odds for the TT-genotype by 35% (OR = 0.65, CI = 0.44–0.94, p = 0.02). *COMT* 24938A/G was associated with plasma glucose levels at S-46 where an increment of a one-unit (10 mmol/L) glucose increased the odds for the GG-genotype by 4% (OR = 1.04, CI = 1.00–1.09, p = 0.05; Table 3). The significant associations for plasma glucose and acute insulin response seemed to persist when adjusted for fat-BMI (data not shown). No significant associations were observed with the other examined metabolic traits.

**Table 3:** Odds ratio (OR) including 95% confidence intervals (CI) for *5HT2A* rs6311 (dominant effect of the A allele, AA & AG *vs* GG), *5HT2C* rs3813929 (effect of the T *vs* C alleles) and *COMT* rs4680 (recessive effect of the G allele, GG *vs* GA & AA) in relation to metabolic quantitative traits.

	*5HT2A*		*5HT2C*		COMT	
	-1438G/A		-759C/T		24938A/G	
Metabolic traits	OR (95% CI)	*P*	OR (95% CI)	*P*	OR (95% CI)	*P*
*S-46 (n = 1570)*						
p-glucose (10 mmol/L)	0.98 [0.94; 1.02]	0.38	1.01 [0.96; 1.07]	0.61	1.04 [1.00; 1.09]	0.05
s-Cholesterol (10 mmol/L)	2.66 [1.11; 6.40]	0.03	1.64 [0.54; 4.98]	0.38	1.23 [0.44; 3.49]	0.69
s-HDL (mmol/L)	0.88 [0.66; 1.16]	0.36	0.86 [0.59; 1.25]	0.43	0.79 [0.56; 1.12]	0.18
Systolic BP (10 mmHg)	0.97 [0.91; 1.03]	0.31	1.00 [0.92; 1.08]	0.93	0.98 [0.90; 1.05]	0.48
*S-49 (n = 551)* 1						
p-glucose (10 mmol/L)	1.74 [0.50; 6.09]	0.38	4.56 [1.13; 18.4]	0.03	0.75 [0.13; 4.32]	0.75
p-insulin (50 pmol/L)	1.13 [0.91; 1.39]	0.27	0.73 [0.51; 1.06]	0.10	1.05 [0.81; 1.37]	0.72
BIGTT-S_I_	0.94 [0.77; 1.15]	0.55	0.94 [0.72; 1.22]	0.64	0.89 [0.69; 1.14]	0.36
BIGTT-AIR	0.98 [0.74; 1.30]	0.88	0.65 [0.44; 0.94]	0.02	1.22 [0.83; 1.78]	0.31
s-Cholesterol (10 mmol/L)	3.29 [0.56; 19.5]	0.19	0.97 [0.10; 10.9]	0.98	0.19 [0.02; 1.94]	0.16
s-Triglycerides (mmol/L)	0.91 [0.77; 1.08]	0.29	1.17 [0.98; 1.39]	0.08	1.01 [0.83; 1.23]	0.93
s-HDL (mmol/L)	1.13 [0.59; 2.16]	0.73	1.32 [0.54; 3.19]	0.54	0.74 [0.32; 1.72]	0.49
s-FFA (mmol/L)	0.96 [0.30; 3.08]	0.95	2.37 [0.49; 11.4]	0.28	1.06 [0.24; 4.74]	0.94
Systolic BP (10 mmHg)	1.02 [0.91; 1.14]	0.73	1.04 [0.91; 1.20]	0.56	0.92 [0.80; 1.06]	0.22

BP = blood pressure, BIGTT-S_I_ = OGTT-derived index of insulin sensitivity, BIGTT-AIR = OGTT-derived index of acute insulin response, FFA = free fatty acids.

1 All values for the S-49 are derived from the OGTT examination and are therefore fasting compared with non-fasting for S-46.

### IGT and T2D

Based on the above findings on glucose homeostasis, we examined *5HT2C* –759C/T and *COMT* 24938A/G in relation to IGT and T2D. The *5HT2A* –1438G/A did not associate with glucose homeostasis, and therefore associations with IGT or T2D were not examined. Results from logistic regression analyses in 162 subjects with IGT or T2D and 308 glucose-tolerant subjects showed corresponding notable estimates, although of borderline significance. Subjects with IGT or T2D had a trend to increase the odds for the T-genotype for *5HT2C* and GG-genotype for *COMT* 24938A/G by 53% (OR = 1.53, CI = 0.90–2.61, p = 0.12) and 48% (OR = 1.48, CI = 0.91–2.41, p = 0.12), respectively compared to glucose-tolerant subjects **(** **Table 4** **)**. Adjustment for fat-BMI did not influence the estimates notably.

**Table 4:** Odds ratio (OR) including 95% confidence intervals (CI) for *5HT2C* rs3813929 (effect of the T *vs* C alleles) and *COMT* rs4680 (recessive effect of the G allele, GG *vs* GA & AA) in relation to IGT and T2D compared to glucose-tolerant subjects before and after adjustment for fat-BMI (kg/m^2^).

	*5HT2C*		COMT	
	-759C/T		24938A/G	
IGT and T2D	OR (95% CI)	*P*	OR (95% CI)	*P*
*S-49 (n = 451)*				
Unadjusted	1.53 [0.90; 2.61]	0.12	1.48 [0.91; 2.41]	0.12
Adjusted for fat-BMI	1.50 [0.83; 2.71]	0.18	1.45 [0.83; 2.55]	0.19

IGT = impaired glucose tolerance, T2D = type 2 diabetes, fat-BMI = fat body mass index.

**Figure 1:**
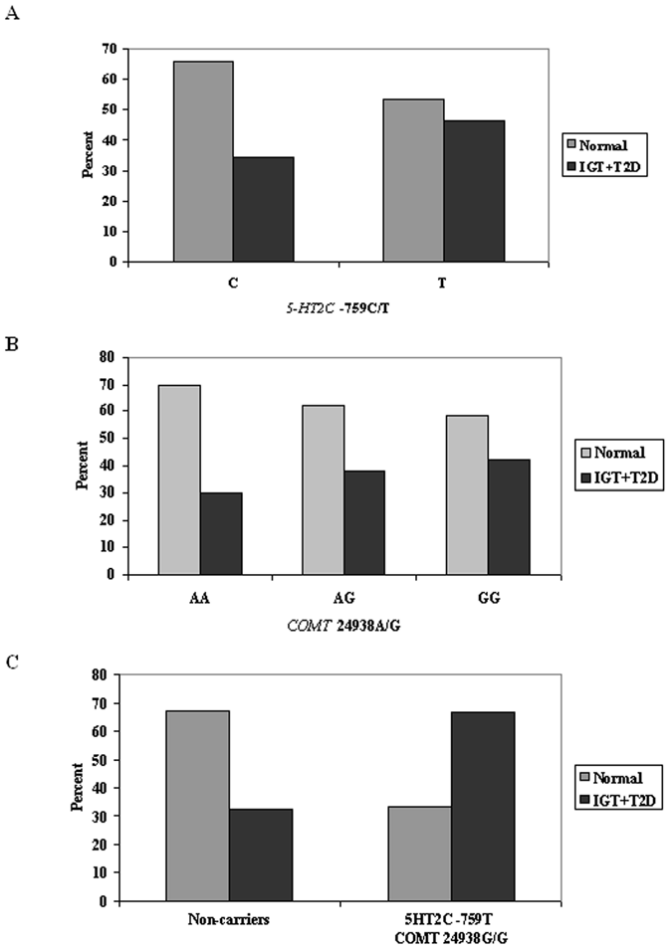
Frequency of IGT or T2D according to *5HT2C* –759C/T, *COMT* 24938A/G and combined genotype analysis in the combined group of obese and randomly selected men. Values are given as percentages. *A:* Frequency of IGT or T2D according to *5HT2C* –759C/T. *B:* Frequency of IGT or T2D according to *COMT* 24938A/G. *C:* Frequency of IGT or T2D according to combination of *5HT2C* –759C/T T-allele and *COMT* 24938A/G GG-genotype. *A:* P-value for χ^2^ = 0.05, *B:* P-value for χ^2^ = 0.06, *C:* P-value for χ^2^ = 0.01.

In total, 46.5% of the subjects with the T-genotype of *5HT2C* –759C/T had IGT or T2D, whereas only 34.2% subjects with the C-genotype had IGT or T2D (χ^2^: p = 0.05), implying a 12.3% increased frequency of the T-allele in the presence of IGT or T2D compared with glucose tolerant subjects **(** **Figure 1A** **)**. *COMT* 24838A/G was also associated with IGT and T2D. In total, 41.9% of the subjects with the GG-genotype had IGT or T2D, whereas only 30.3% subjects with the AA genotype had IGT or T2D (χ^2^: p = 0.06), implying 11.6% increased frequency of the GG-genotype, in the presence of IGT or T2D **(** **Figure 1B** **)**.

### Combined effect of 5HT2C and COMT

Based on the present findings on glucose homeostasis, IGT and T2D for *5HT2C* and *COMT*, we examined the combined genotype association of the minor risk genotypes of *5HT2C* and *COMT* in relation to obesity and related metabolic traits. In total, 58 (4.7%) subjects were carriers of both *5HT2C* –759T and GG-genotype of *COMT* 24938A/G. Analysis of the combined genotype has been conducted only in S-46, because of a low number of carriers in S-49 (n = 12).

We observed significant associations between carriers of the combined risk genotype, all obesity phenotypes and total serum cholesterol. A two-unit increase in BMI was associated with increased odds for being carrier of the combined genotype by 12% (OR = 1.12, CI = 1.03–1.21, p = 0.006) **(** **Table 5** **).** Likewise a two-unit increase in fat-BMI increased the odds for being carrier of the combined genotype by 22% (OR = 1.22, CI = 1.08–1.38, p = 0.002). Each increment of five cm in waist circumference increased the odds for being carrier of the combined genotype by 13% (OR = 1.13, CI = 1.04–1.22, p = 0.003). Lastly, a one-unit (10 mmol/L) increase in total serum cholesterol increased the odds for being carrier of the combined genotype 5.6 fold (OR = 5.60, CI = 0.99–31.4, p = 0.05). Adjustment for fat-BMI showed that the associations between the combined risk genotype, BMI, waist circumference and total serum cholesterol were in part explained by an effect of fat-BMI.

**Table 5:** Combined genotype analyses. Odds ratio (OR) including 95% confidence intervals (CI) for *5HT2C* T-allele (rs3813929) and *COMT* GG-genotype (rs4680) in relation to metabolic quantitative traits before and after adjustment for fat-BMI (kg/m^2^).

	*5HT2C –759T COMT 24938G/G*			
	Unadjusted		Adjusted for fat-BMI	
Obesity phenotypes	OR (95% CI)	*P*	OR (95% CI)	*P*
*S-46 (n = 1570)*				
BMI (kg/m^2^)^1^	1.12 [1.03; 1.21]	0.006	1.01 [0.61; 1.69]	0.96
Fat-BMI (kg/m^2^)^1^	1.22 [1.08; 1.38]	0.002	**−**	**−**
Waist (cm)^2^	1.13 [1.04; 1.22]	0.003	1.13 [0.87; 1.45]	0.36
**Metabolic traits**				
p-glucose (10 mmol/L)	1.06 [0.98; 1.14]	0.15	**−**	**−**
s-Cholesterol (10 mmol/L)	5.60 [0.99; 31.4]	0.05	4.90 [0.82; 29.2]	0.08
s-HDL (mmol/L)	0.76 [0.37; 1.56]	0.45	**−**	**−**
Systolic BP (10 mmHg)	1.09 [0.94; 1.26]	0.24	**−**	**−**

BMI = body mass index, fat-BMI = fat body mass index, BP = blood pressure.

p- = plasma, s- = serum.

1 Per two-unit increment of BMI or fat-BMI.

2 Per five cm increment of waist circumference.

Carriers of the combined risk genotype were also associated with IGT or T2D, where 66.7% of the carriers had IGT or T2D compared with only 32.6% in non-carriers (χ^2^: p = 0.01) **(** **Figure 1C** **)**.

## Discussion

The present study revealed that the examined SNPs of *5HT2A* and *5HT2C* did not associate with obesity, but *COMT* 24938A/G was associated with fat-BMI. Associations were observed between the examined SNPs, glucose homeostasis, serum cholesterol level and a surrogate measure of acute insulin response. Combined genotype analysis of *5HT2C* –759C/T and *COMT* 24938A/G revealed associations in relation to BMI, fat-BMI, waist circumference and serum cholesterol levels, and were partly explained by a possible mediating effects of fat-BMI. *5HT2C* –759T and the GG-genotype for *COMT* 24938A/G showed an increased frequency in subjects with IGT or T2D. The combined genotype analyses accentuated these effects and showed that carriers of the combined risk genotype had increased frequency of IGT or T2D. The present results have not been replicated and, given the experience of complex trait genetics, warrant caution in interpretation.

The present population-based cohort study of Danish Caucasian men where the controls were randomly selected from the same population in which the cases were identified effectively prevents population stratification. The availability of several repetitive measurements in the same individuals have given the unique opportunity of analyzing and comparing a panel of specific obesity-related phenotypes covering BMI, fat-BMI, waist circumference and metabolic traits at two different ages. The samples may seem small for a genetic association study; however, this apparent limitation of the study is counteracted by the fact that the control group represents approximately 200 times the size of the randomly selected control group originally identified at the draft board examination. The obese participants therefore were representing the most extreme range of the obesity-related phenotypes in this population at both surveys except for the possible effects of differential attrition of the population during the follow-up. The statistical efficiency of the current sampling design, when analyzed as dichotomized case-control studies, corresponds to about half what would be achieved by investigating the full cohort. Keeping the phenotypes as quantitative variables in the analyses, the efficiency is considerably higher as reflected in the fairly narrow CIs, which means that we thereby have narrowed down the likely true OR's that could have given rise to the observed ORs. Thus, owing to the sampling design of the obese participants with massive enrichment of the right tail of the BMI distribution it is possible to demonstrate stronger associations in our cohort compared to already published studies examining obesity-related genotype-phenotype associations.

By nature, our findings are exploratory, and the significant *P*-values were therefore not corrected for multiple hypotheses testing. Estimating the false discovery rate by the method of Benjamini and Hochberg [42], the adjusted *P*-value for 68 dependent tests would be 0.011, which implies that all significant associations, except for the combined genotype analyses in relation to obesity, would be false-positive. None of the exploratory results would reach the enhanced significance criterion under application of the conservative Bonferroni correction for an overall type I error rate of 0.05 (one false-positive per 20 tests), which would result in a significance threshold of 0.0007. The examined phenotypes may be inter-correlated to various extents, but according to a recent twin study [43], there is little common underlying genetic or shared environmental aetiology behind these correlations, which justifies the separate analysis of each of the phenotypes in the present study.

Although, we have avoided sex-related confounding, which otherwise may have an impact on the genetic variation of body composition, the results suggest that also the role of the gene variants should be examined in women. The –759T allele is functional and has been associated with increased promoter activity compared with the wild type –759C allele [5]. There is indication of sex-related effects and one study has reported higher frequency of the C-allele in obese women, which was associated with weight loss resistance in heterozygote women suggesting that women are more prone to serotonin-induced weight changes [44]. Only one recently published study has examined the effect of –759C/T of the *5HT2C* receptor gene on obesity and T2D in a population-based study design [45]. Results from the study of 315 Greek men and women showed a positive association between *5HT2C* rs3813929 and T2D, but not with BMI. However, in the same study, the *5HT2C* –759T allele frequency was 10.7% lower in diabetic individuals compared to glucose tolerant individuals, which is in discordance with our finding of a 12% increased frequency of the T-allele in men with IGT or T2D (accentuated by combined genotype analysis). The present discrepancy may be ascribed to a high mean age of the Greek subjects and a small study population not enriched on obesity.

*COMT* has been studied intensively in relation to several reward-motivated behaviours such as development of diet-induced obesity [27], [46]. It is known that the G-allele of *COMT* rs4680 is associated with low COMT activity of soluble COMT, thus conferring slow detoxification of neurotransmitters [47] such as the degradation and inactivation of dopamine [26], [46]. We found an association between *COMT* 24938A/G and fat-BMI, and borderline with waist circumference, which is in accordance with the suggested role of *COMT* polymorphisms in obesity [24], [27], [48] and in line with results from a recently published population-based study showing an association between *COMT* 24938A/G and body fat distribution measured as waist-hip ratio [48]. In a recent randomized controlled trial, no association between *COMT* 24938A/G and obesity was observed, yet a significant association between a different *COMT* SNP (rs4818) and BMI was reported [27]. However, because COMT is affecting both the degradation of noradrenaline and dopamine, a decreased activity of the gene product will enhance the post-receptor effects of these neurotransmitters. In this context, it is interesting that re-uptake inhibition on these two systems, that will tend to have the same effect, is an effective way to produce a weight loss in humans [12], [25].

While, there is increasing appreciation for the role of serotonin, noradrenaline and dopamine pathways in energy homeostasis and hence as targets for development of effective anti-obesity agents [8], [49], [50], we did not detect associations of significant importance with obesity. At first step, our results suggested that *5HT2C* –759C/T and *COMT* 29438A/G is associated with IGT and T2D, but partly independent of obesity. However, our results also implied that the combined genotypes of *5HT2C* –759C/T and *COMT* 29438A/G may have an impact on obesity and thus, in this way, on the development of IGT or T2D. The present and potential discrepancies between studies underline the continuing challenges of studies with different populations with different characteristics and pathophysiological and life-style related characteristics that may modify the effects of the examined gene variants.

Our findings lend further support to the involvement of serotonin, noradrenaline and dopamine in energy and glucose homeostasis, and hence on the risk of obesity and T2D, in particular when combined genotype associations are explored. The results underscore the need for additional research in order to replicate the results and to identify the complex interplay between the examined psychophysiological genes that may further characterize their functionality in energy homeostasis.
